# Desiccation as a suitable alternative to cold-storage of phyllosphere samples for DNA-based microbial community analyses

**DOI:** 10.1038/s41598-024-82367-x

**Published:** 2025-02-04

**Authors:** Emily Smenderovac, Karelle Rheault, Marie-Ange Moisan, Caroline Emilson, Élodie Brazeau, Marie-Josée Morency, Patrick Gagné, Vincent Maire, Erik Emilson, Lisa Venier, Christine Martineau

**Affiliations:** 1 Great Lakes Forestry Centre, Natural Resources Canada, ontario, Canada; 2 Present Address: Laurentian Forestry Centre, Natural Resources Canada, Québec, Canada; 3 Université du Québec à Trois-Rivières, Québec, Canada

**Keywords:** Environmental sciences, Community ecology, Ecological genetics, Ecosystem ecology, Forest ecology, Microbial ecology, Molecular ecology

## Abstract

The study of microbial communities of the plant phyllosphere in remote locations using DNA-based approaches is limited by the challenges associated with their preservation in the field and during transportation. Freezing is a common DNA preservation strategy, but it may be unsuitable for leaf samples, or inaccessible in some locations. Other methods such as desiccation, ethanol or commercial preservatives are potential alternative DNA preservation methods for ambient temperature storage. In this study, we assessed the efficacy of desiccation (with silica gel packs), and of three preservation solutions (95% ethanol, RNAlater, LifeGuard) for the preservation of epiphytic phyllosphere communities of *Populus tremuloides* and *Picea glauca* at ambient indoor temperature (21 °C) for up to three weeks. We assessed effects on DNA concentration and quality and used metabarcoding to detect changes in bacterial and fungal communities between treatments over time. A secondary study was conducted on leaves of *Populus grandidentata* to further test the ability of the desiccation treatment to resolve differences between sampling sites. Silica gel packs were identified as effective ambient temperature preservative of phyllosphere bacterial and fungal communities. There were some changes in the communities compared to immediate extraction due to this treatment, but these changes did not affect the ability to distinguish tree species and sampling locations. Overall, our study supports the use of silica gel pack short term preservation at ambient temperature for phyllosphere samples intended for DNA-based microbial community analyses.

## Introduction

The study of microbial communities colonizing the aerial of plants (i.e., the phyllosphere) with DNA based methodologies is important for our understanding of the microscale ecology of forests. Yet, these communities remain less commonly assessed than those from soils despite the recognized importance of these microbial associations to plant health^1,2^. Sampling and preserving phyllosphere samples for microbial community analyses can be difficult due to the close associations of microorganisms with their hosts^3^. Phyllosphere samples for microbial community analyses are therefore usually kept cold and processed as soon as possible after collection^4–6^. The cold storage and short timeline requirements make the execution of large or widely distributed phyllosphere microbiome sampling logistically challenging. Methods used for the recovery of phyllosphere microbial communities vary based on the component of the community that is being targeted: while endophytic communities are located within plant tissues, epiphytic communities colonize plant surfaces. Epiphytic microbial communities are typically recovered by washing the surface of leaves in a buffer solution, sometimes including a sonication step followed by filtration or centrifugation^7^. The epiphyte extraction process can interact with preservation treatments unfavourably: damage to the structural integrity of leaf tissue caused by some treatments can lead to contamination of samples with plant and endophyte DNA, interfering with the amplification of epiphytic DNA^8,9^. As an example, freezing, a common method for the preservation of microbial community samples, can lead to cell damage that increases the introduction of plant DNA into samples^10^. This is a greater concern in the more commonly encountered freezing temperatures of -20 °C to -30 °C, which freeze samples more slowly, and can introduce cell-wall disrupting ice crystals into samples. It is unknown to what extent different preservation methods affect the amount of plant and endophytic DNA in the resulting extracts.

Validation of preservation strategies other than freezing and/or cooling can add flexibility to existing local sampling regimes, as well as enable phyllosphere sampling in situations where it was previously impossible. It also reduces the likelihood of sample losses due to shipping interruptions or delays^11^. Some preservation methods are suitable for higher temperature storage: Desiccation (drying) has been identified as a promising approach for preserving microbial communities in the short to long term^5,12,13^ and has even been used for phyllosphere samples in a previous study^14^. Desiccation is also commonly used for the preservation of samples for plant DNA analyses^4^ and could therefore be a suitable approach for the simultaneous study of microbial and host plant DNA. Some commercial products, such as RNAlater solution (Invitrogen) could also be effective for the preservation of samples at ambient temperature, but these are typically designed for tissue preservation and have not been extensively tested for the preservation of plant-associated microbial communities^15,16^. Some studies have indicated that ethanol can be used to maintain DNA integrity at ambient temperatures (as it is regularly employed in arthropod studies)^4,17^. Overall, there is little research on which methods are suitable for preserving plant samples for epiphytic microbial community analyses when cold storage is not an option.

In this study, we evaluate the effectiveness of desiccation with silica gel packs and of three storage solutions for the preservation of phyllosphere samples at ambient indoor temperature (21 °C) for DNA-based microbial community analyses. Bacterial and fungal diversity and community structure as well as contamination of extracts with plant DNA obtained with these treatments were compared to those of optimal (i.e., DNA extraction on the day of sampling) and standard (i.e., freezing) preservation conditions. These treatments were applied to needles of a coniferous species (*Picea glauca* (Moench) Voss) and leaves of a deciduous species (*Populus tremuloides* (Michx.)) which are both abundant in the boreal forests of Canada. We also further assessed the impact of the desiccation treatment on the detection of site-level differences for the deciduous species (*Populus grandidentata* (Michx.)). The information provided will better inform planning for microbial phyllosphere ecology projects, potentially expanding the ability to sample in remote and semi-remote contexts, and increasing the window of time for phyllosphere sample shipping and processing.

## Methods

### Sample collection for assessing effectiveness of five preservation methods

Samples for this experiment were collected at the Valcartier Forestry Research Station in Saint-Gabriel-de-Valcartier (Québec, Canada). All samples were collected on crown land as part of federal government research objectives. Sample collections were compliant with international and domestic legislation. Two different sample types were collected: white spruce (*Picea glauca*) needles and, trembling aspen (*Populus tremuloides*) leaves. Bulk samples of leaves and needles were taken from multiple branches randomly selected on a single tree using a pruner. Needles were only taken from the two last generations of twigs to reduce between sample variability. Large numbers of individual leaves (aspen) or 5 cm twigs (spruce) were recovered, placed in plastic bags and stored in a cooler with ice. Once in the lab, all samples were stored at 4 °C until further processing.

Bulk samples were split into sets of four leaves (aspen), or four twigs (spruce) per sample. We tested immediate extraction, freezing at -30 °C for three weeks (Freeze) and four ambient indoor temperature (21 °C) preservation methods: Desiccation (10 g Dry & Dry silica gel packs) (Dry); LifeGuard solution (QIAGEN) (LG); RNAlater solution (Invitrogen, Thermo Fisher) (RL); 95% ethanol (EtOH). Nine samples from each tree species were prepared to account for three replicates at three time-points: 1 week, 2 weeks, and 3 weeks of incubation. Three replicate samples were prepared for immediate extraction and for the Freeze treatment, for which there was a single time point.

For the desiccation treatment, four leaves (or twigs) were added to a plastic bag containing two 10 g Dry & Dry silica gel packs. For the samples stored in preservation solutions (i.e. RNAlater, LifeGuard, ethanol), 4 ml of solution was first transferred to 5 ml tubes. For *P. tremuloides* samples, four leaves were stacked, veins down, and folded vertically along the midrib, making sure that the top of the leaf was facing out. The stack of folded leaves was then rolled starting from the petiole, and the resulting roller was inserted into the 5 ml tube with the main rib facing the top of the tube. The tube was delicately shaken to ensure that the liquid completely covered the leaves. For *P. glauca* samples, four twigs of 4–5 cm were put in the tubes. All the samples were then incubated in a dark growth chamber under stable conditions at 45% humidity and 21 °C (to approximate ambient indoor temperature as well as summer air temperature in the shade) and removed after 1, 2 or 3 weeks of incubation. Samples subjected to freezing were stored in plastic bags containing four leaves (or twigs) and placed in a -30 °C freezer for 3 weeks.

## Sample collection for assessing the detection of site level variability of phyllosphere communities

A second sampling campaign was conducted with the objective of assessing the effect of the desiccation preservation on the detection of differences in epiphytic microbial communities among sampling sites for a deciduous tree species, bigtooth aspen (*Populus grandidentata*). Samples were collected on public municipal park land as part of federal government research objectives. Sample collections were compliant with international and domestic legislation. This species was selected for this assessment because of its later senescence compared to *P. tremuloides*, enabling a fall sampling event. Bulk samples of leaves of *P. grandidentata* were collected from three individuals per site, at three sites (i.e., Base plein air de Ste-Foy, 46.79037, -71.32690; Parc des Chutes de la Chaudière, 46.71794, -71.28260; St-Étienne de Lauzon, 46.66272, -71.29950) following the procedure described above for *P. tremuloides*. Leaves from each individual tree were distributed in the different treatments (5 leaves/treatment): immediate extraction, freezing for three weeks (-20 °C) and desiccation (10 g Dry & Dry silica gel packs) for one or three weeks. Storage conditions were as described above for the desiccation and freezing treatments.

## DNA extraction

To extract epiphytic phyllosphere DNA, the leaves (or twigs) were transferred (with the storage solution, if applicable) using sterile forceps into 50 ml tubes containing 25 ml of PBS 1 × (8 g NaCl, 0.2 g KCl, 1.44 g Na_2_HPO_4_, 0.24 g KH_2_PO_4_, pH 7.4, 1 L distilled water) + Tween 20 (0.1%). For dried or frozen leaves (or twigs), a small volume of PBS + Tween buffer was also poured in the plastic bag, shaken, and poured back in the 50 ml tubes to make sure all cells from the leaf surface were collected. The tubes were then vortexed for three minutes at maximum speed. The leaves (or twigs) were removed from the tubes, keeping as much liquid as possible in the tubes. Then, the tubes were centrifuged for 20 min at 4000 g and 4 °C. The supernatant was carefully removed by pipette, leaving about 2 ml of liquid. The pellet was delicately resuspended, transferred to a 2 ml tube and centrifuged for 1 min at 15,000 g. The supernatant was discarded and 800 µl of CD1 solution (QIAGEN DNeasy PowerSoil Pro kit) was added to resuspend the pellet. The content of the tube was transferred to a PowerBead tube from the kit followed by DNA extractions following the manufacturer’s instructions with the QIAcube system (QIAGEN).

## DNA quantification, sequencing and bioinformatics

DNA quantification, quality assessment, library preparation for the 16S rRNA gene (bacterial communities) and ITS2 region (fungal communities), Illumina MiSeq sequencing and, bioinformatics were performed as described in Smenderovac et al.^13^. Briefly, bacterial communities were amplified using primers 515 F-Y (5′-GTGYCAGCMGCCGCGGTAA-3′) and 926R (5′- CCGYCAATTYMTTTRAGTTT-3′)^18,19^ and The ITS2 region of the fungal ribosomal DNA was amplified using the primer set ITS9F (5′-GAACGCAGCRAAIIGYGA-3′) and ITS4R (5′-TCCTCCGCTTATTGATATGC-3′)^19,20^. Metabarcoding libraries for each sample and amplicon were performed and pooled after normalization of the DNA concentrations. Sequence libraries were subjected to paired end sequencing (2 × 300 bp) at the Centre de recherche du CHU de Québec-Université Laval. Bioinformatics was performed in QIIME2 (version 2021.8)^21^, using DADA2 (denoised paired command algorithm)^22^ for ASV selection. The taxonomic assignment of ASVs was done using the SILVA 138 database for the 16 S rRNA gene^23,24^ and the UNITE database (version 8.0) for the ITS2 region^25^. The Illumina sequence data generated in this study were deposited in the NCBI Sequence Read Archive and are available under the project number PRJNA982550 with the accession numbers SRR29206223 to SRR29206306 for 16 S sequences, and SRR29206861 to SRR29206944 for ITS sequences.

## Reagent costs

Costs of each preservation agent were acquired from Amazon, ThermoFisher, QIAGEN and Fisher Scientific on April 23, 2024, estimates of costs were all calculated in Canadian Dollars using the exchange rate on that day (.73CAD/USD). Shipping and any additional costs were not assessed.

### Statistical analysis

All statistical analyses were performed in R version 4.3.1 (2023-06-16 ucrt)^26^ using tidyverse v2.0.0^27^ for data transformations while visualizations were performed with ggplot2 v3.5.0^28^ and ggpubr v0.6.0^29^. Defined contrasts were used to compare responses of preservation methods at each week of extraction to immediate extraction. Proportion of reads assigned to chloroplast and to mitochondria was extracted from the unfiltered ASV table and used as a proxy for the contamination of epiphytic DNA extracts with plant DNA. All other ASV analysis were performed after filtering out ASVs assigned to chloroplast and mitochondria from the ASV table. Communities were rarefied to the 15th percentile of sample reads in each dataset and diversity indices were calculated using vegan v2.6.8^30^ as in Smenderovac et al.^13^. ASV richness was used for both bacterial and fungal communities, while Shannon and inverse Simpson’s indices were used for bacterial communities only as large variations in fungal ITS copy numbers^31^ could impact these metrics. An analysis of variance (ANOVA) model using the aov function in R was applied to assess the effect of preservation treatment on DNA concentration, DNA quality, Shannon-Wiener diversity index, Inverse Simpson’s diversity index and ASV richness. Normality of residuals for all ANOVA tests were examined visually and using Shapiro normailty tests to verify the appropriateness of the approach. Results were assessed in the context of Holm-adjusted p-values as well as unadjusted p-values, at an alpha of *p* < 0.05 to provide different levels of confidence for observed effects. Changes in community structure were tested with beta-dispersion and permutational analysis of variance (PERMANOVA) (using the betadisper and adonis2 functions in vegan) using Aitchison distances (i.e., center-logged-ratio transformed datasets assesed with Euclidean distance using the adonis2 function)^30^, and visualized with ordination using PCA of Aitchison distances. Individual ASV responses to treatments were assessed with the ancombc2 function in the ANCOMBC package v2.2.1^32^. For the comparison of the effect for dessication treatments on the detection of sampling location effect, between-site Aitchison distances were directly compared to within-site distances. Detection of site effects on alpha diversity metrics were evaluated with a mixed-effect ANOVA using the aov function in base R^26^, and overall community composition effects were evaluated with mixed effect PERMANOVA, with the model using an interaction between treatment and site using the adonis2 function of the vegan package^30^.

## Results

### Comparison of multiple ambient temperature preservation methods for phyllosphere samples

#### Reagent costs

**Table 1:** Per-sample costs of sample reagents for the preservation of phyllosphere samples at ambient temperature for DNA-based community analyses. Costs are estimates based on pricing acquired on 2024-04-23.

Treatment	Cost per unit ($CAD)	Unit size	Number of samples per unit	Additional Cost per sample ($CAD)	Supplier Information
Desiccant (Dry & Dry silica pouches)^1^	78.010	100 packs	25	1.56	Amazon
LifeGuard^2,3^	3456.164	1000 mL bottle	250	13.82	QIAGEN, cat# 12,868 − 1000
RNAlater^2^	704.000	500 mL bottle	125	5.63	ThermoFisher, cat# AM7021
95% Ethanol^2^	170.150	4000 mL bottle	1000	0.17	Fisher Scientific, cat# LC222054

^1^ Two packs of desiccant were used per sample.

^2^ Four mL of preservation solution were used per sample.

^3^ Pricing for Qiagen LifeGuard is from 2023, as product was no longer offered in 2024.

Desiccation and 95% ethanol were the most inexpensive options, all being under $2CAD a sample, while LifeGuard and RNA later added more than $5 a sample to overall costs (Table 1).

### DNA concentration and quality

DNA recovery was generally low across all samples for epiphytic phyllosphere samples in both *Picea glauca* and *Populus tremuloides* ranging between 0 and 8.2 ng/µL.

**Fig. 1:**
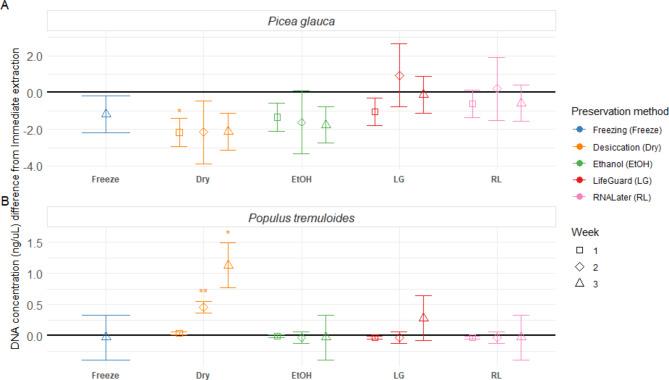
Preservation method effect size on DNA concentration (ng/mL) compared to immediate extraction for (**A**) *P. glauca* and (**B**) *P. tremuloides* phyllosphere samples. Points display the estimated effect (difference from immediate extraction, black line) introduced by the preservation method, and error bars represent the standard error of the estimate (*n* = 3). For each preservation method, the incubation periods are displayed in order (week one to three, from left to right), except for freezing, which was only preserved for three weeks. Significant differences from immediate extraction are indicated with an asterisk; * represents significance at *p* < 0.05, and ** represents significance at a holm-adjusted *p* < 0.05.

The range of DNA concentrations for the immediate extraction treatment was 0.02–2.71 ng/µL. Desiccation was the only treatment with significant differences from immediate extraction, with lower DNA concentrations in *P. glauca* samples at week one, and higher concentrations in *P. tremuloides* samples at week two and three (Fig. 1; Table 2). Ethanol, freezing, LifeGuard and RNALater treatments were not significantly different from immediate extraction (Fig. 1; Table 2). Effect of treatments on DNA quality was not assessed, as data was missing for *P. tremuloides* desiccation and ethanol treated samples as all the extract was used for concentration assessment and sequencing. Data for remaining samples was unreliable due to the relatively low DNA concentrations.

### Chloroplast and mitochondrial DNA contamination

**Fig. 2:**
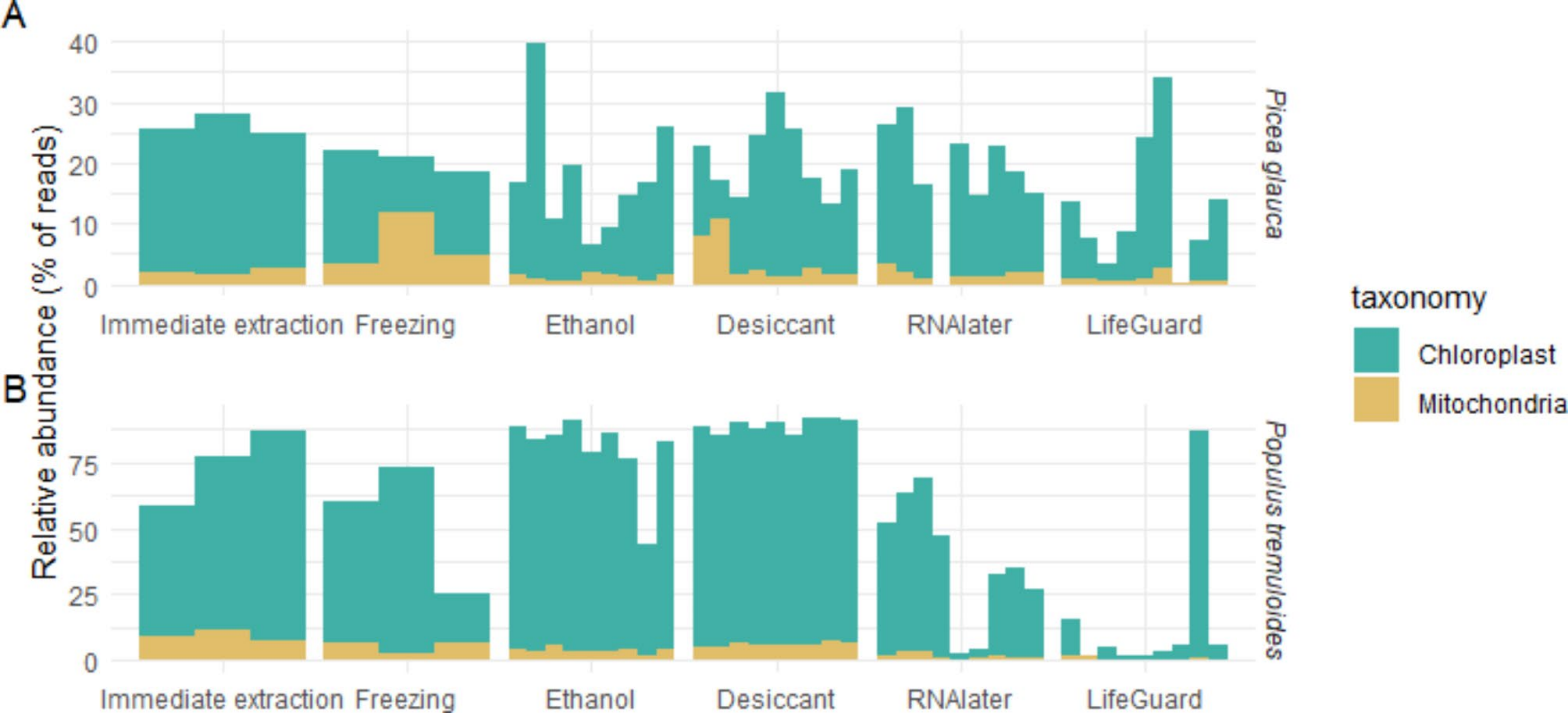
Percent of total metabarcoding 16 S amplicon reads identified as mitochondrial or chloroplast DNA for (**A**) *P. glauca* and (**B**) *P. tremuloides* phyllosphere samples.

Co-extraction and amplification of chloroplast and mitochondrial DNA from plants was not a significant issue in most of the *P. glauca* samples, with the proportion of reads from plant chloroplasts and mitochondria representing approximately 25% of the total reads, on average. All preservation methods generally decreased the proportion of plant DNA amplified from *P. glauca* extractions (Fig. 2). In *P. tremuloides samples* the proportion of co-amplified plant DNA was higher (approximately 75% of reads on average) and the desiccation and ethanol treatments further increased this proportion, while RNAlater, LifeGuard and freezing either reduced or had similar proportions of plant DNA as immediate extraction (Fig. 2).

### Phyllosphere epiphytic communities

#### Alpha diversity

**Fig. 3:**
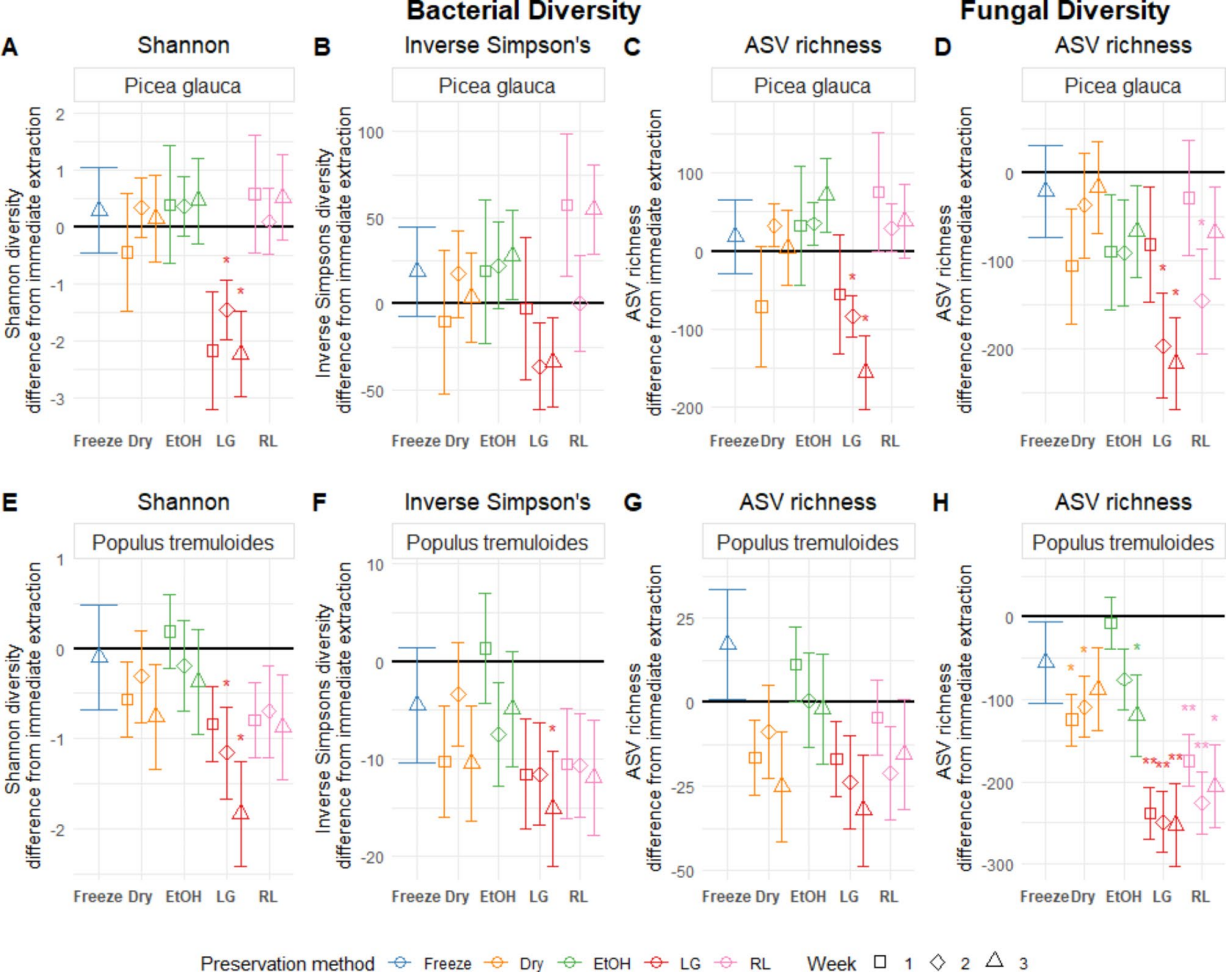
Preservation method effect sizes on bacterial Shannon’s index, inverse Simpson’s index, ASV richness and fungal ASV richness compared to immediate extraction for *Picea glauca* and *Populus tremuloides* phyllosphere samples. Points display the estimated effect (difference from immediate extraction, black line) introduced by the preservation method, and error bars represent the standard error of the estimate (*n* = 3). For each preservation method, the incubation periods are displayed in order (week one to three, from left to right), except for Freezing, which was only preserved for three weeks. Significant results are indicated with an asterisk; * represents significance at *p* < 0.05, and ** represents significance at a holm-adjusted *p* < 0.05. Panels show the following: (**A**) Bacterial Shannon diversity in *P. glauca*, (**B**) Bacterial inverse Simpson’s diversity in *P. glauca*, (**C**) Bacterial ASV richness in P. glauca, (**D**) Fungal ASV richness in *P. glauca,* (**E**) Bacterial Shannon diversity in *P. tremuloides*, (**F**) Bacterial inverse Simpson’s diversity in *P. tremuloides*, (**G**) Bacterial ASV richness in *P. tremuloides*, (**H**) Fungal ASV richness in* P. tremuloides*.

Of the ambient temperature preservation methods, desiccation and ethanol had the highest similarity to immediate extraction for preserving bacterial and fungal diversity (Fig. 3; Table 2). The LifeGuard treatment had the strongest impact on bacterial alpha diversity, with lower bacterial Shannon and inverse Simpsons index at week three in both tree species, lower inverse Simpson index at week three in *P. tremuloides*, and lower richness at weeks two and three in *P. glauca*. The RNAlater treatment only had a significant effect on the inverse Simpson index of *P. glauca* after three weeks of preservation), with higher values detected. No significant effects of the freezing, desiccation and ethanol treatments on bacterial alpha diversity were detected.

There was some decreased fungal richness in *P. glauca* for all ambient temperature treatments when compared to immediate extraction, but the effect was not significant for the ethanol and desiccation treatments at any time point, while the LifeGuard and RNAlater treatments led to lower richness in weeks two and three (Fig. 3). Fungal richness in *P. tremuloides* samples was more affected by the ambient temperature preservation treatments, with LifeGuard and RNAlater resulting in large significant decreases to richness compared to immediate extraction for all time points. Desiccation had significantly lower richness than immediate extraction in weeks one and two, and ethanol had significantly lower richness in week three in *P. tremuloides* samples (Fig. 3). No effect of freezing on fungal richness was detected for either *P. glauca* or *P. tremuloides*. There was no clear temporal trend on the bacterial and fungal diversity indices detected for any treatment except LifeGuard, which showed some decreases in diversity over time (Fig. 3).

### Community composition

**Fig. 4:**
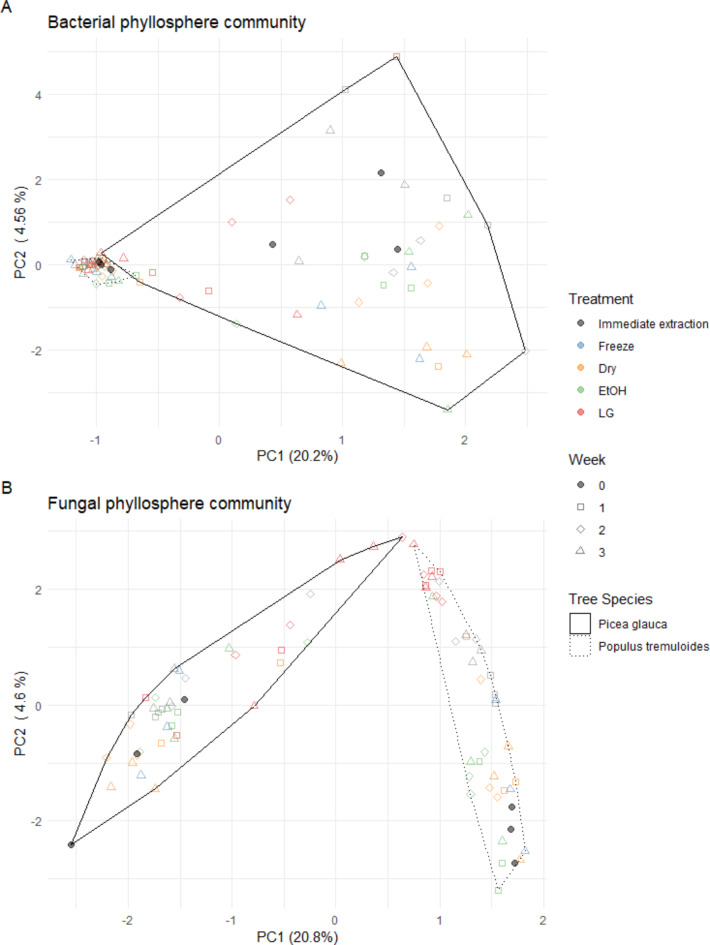
PCA of Bacterial and fungal phyllosphere communities based on Aitchison distances of ASVs for phyllosphere samples subjected to different storage conditions over time. (**A**) Ordination for bacterial communities for all preservation methods for the tree species at all time points, (**B**) Ordination for fungal communities for all preservation methods for the tree species at all time points.

Sample differences (i.e. *P. glauca* needles vs. *P. tremuloides* leaves) were the largest influence on the bacterial (R2 = 0.155, F.value = 0.147, *p* = 0.001) and fungal (R2 = 0.183, F.value = 0.182, *p* = 0.001) community structure, and observation of these differences was not impacted by preservation (Fig. 4). In fact, no significant effect of the preservation treatments to overall community structure were detected compared to immediate extraction, aside from a significant (*p* < 0.05) change in bacterial community structure for the ethanol week three treatment on *P. glauca* needles. Some visible groupings of samples by preservation treatment within each species were observed in ordinations for both bacterial and fungal epiphytic phyllosphere communities, especially for the LifeGuard treatment, but these differences represented a negligible portion of overall community variance (Fig. 4).

**Fig. 5:**
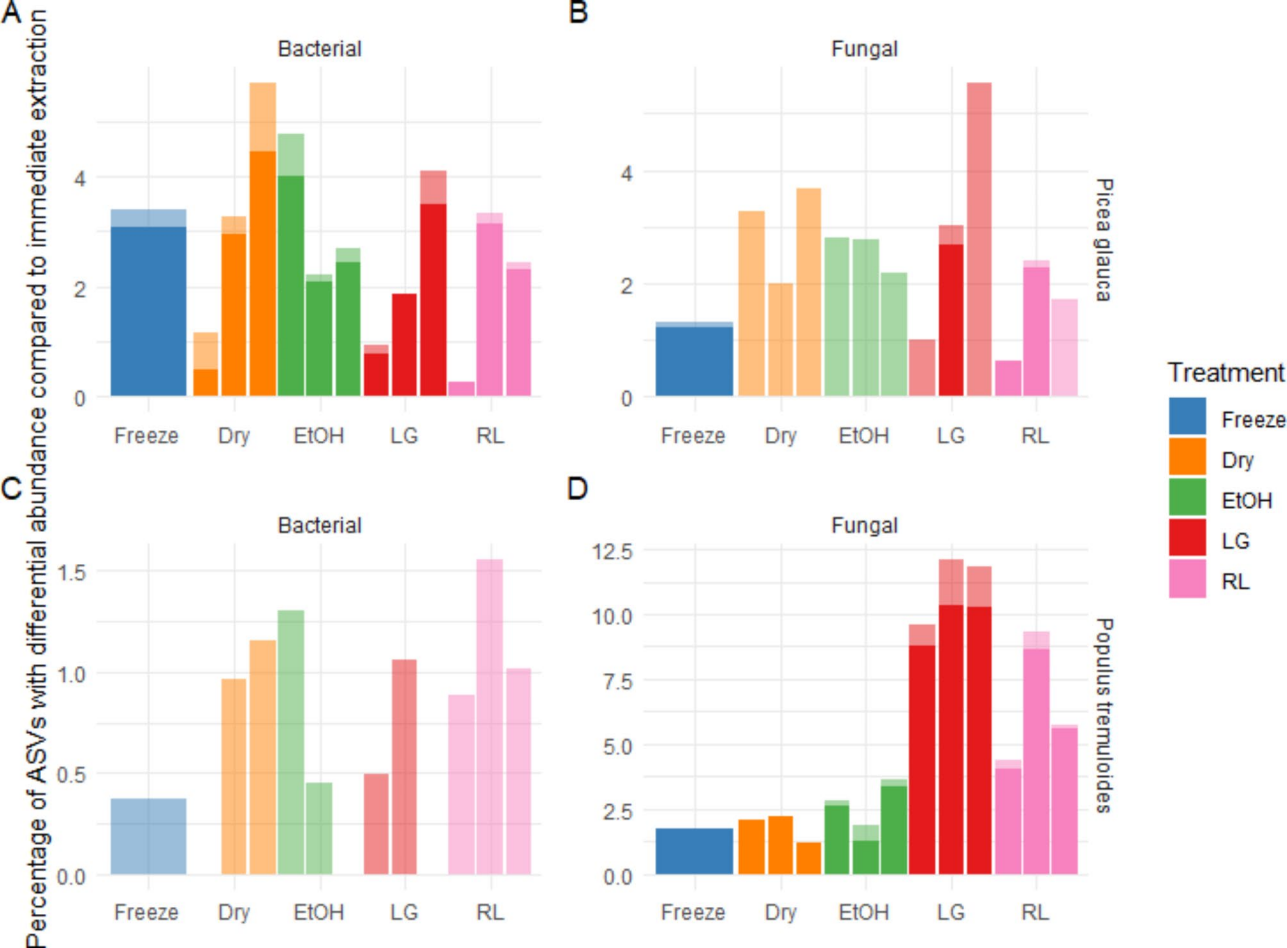
Percent of ASVs in the epiphytic phyllosphere with significant differential abundance compared to immediate extraction. Lighter colors represent the percentage of organisms that had an abundance above 1% in either immediate extraction, or the preservation method, the remaining dark portion of the bar represents organisms in the rare biosphere (< 1% relative abundance). Preservation methods are distinguished using colors and weeks are ordered 1–3 from left to right, except for Freezing, which was only tested on week three. Panels display the following: (**A**) *P. glauca* Bacterial ASVs, (**B**) *P. glauca* Fungal ASVs, (**C**)* P. tremuloides* Bacterial ASVs, (**D**) *P. tremuloides* Fungal ASVs.

All preservation methods generally led to comparable percentages of bacterial ASVs that were differentially abundant compared to immediate extraction for both tree species. A similar trend was observed for fungal ASVs in *P. glauca* (Fig. 5). However, in *P. tremuloides* samples, the LifeGuard amd RNAlater treatments led to higher numbers of differentially abundant fungal ASVs (Fig. 5).

After one week of storage, desiccation caused large changes (> 10% of the relative abundance) in the relative abundance of a few plant-associated taxa (*Bradyrhizobium*, *Massila*) and generalist taxa (*Hymenobacter*, *Ramularia*) (Table S1). These changes were observed only in *P. glauca* samples. Ethanol treatment resulted in both large (> 10%) and small (< 10%) changes in relative abundances of a higher number of taxa than the other treatments after one week of storage. These changes mostly consisted of increases in the relative abundances of *Massila* (endophyte) and of generalist soil taxa (*Acidiphilium*, *Sphingomonas* and *Terriglobus*) and a decrease in relative abundance of *Amnibacterium* (endophyte). LifeGuard and RNAlater led to fewer taxa impacted in the first week, and most changes were observed in *P. tremuloides* samples. This included decreases in the relative abundance of the plant pathogens *Coniothyrium* and *Taphrina* in both treatments. For the RNAlater treatment, the plant associated taxa *Amnibacterium* and *Pantoea* also showed a large (> 10%) increase and decrease, respectively.

After two weeks, important changes were observed in the LifeGuard samples, with decreases in the relative abundance of *Coniothyrium*, *Endoconidioma*, *Microcyclospora*, *Setomelanomma*, *Sphaerulina*, and *Taphrina* and increases in *Sphingomonas* and *Gibberella*. All the other treatments showed similar or lower numbers of impactful taxa compared to week one, but some of the impacted taxa were not the same (Table S1). Most changes observed in week two occurred in *P. tremuloides* samples.

In week three, changes in the relative abundance of several taxa were detected in both the desiccation and LifeGuard treatments, while fewer taxa were impacted by the freezing, ethanol and RNAlater treatments. Desiccation mostly impacted *P glauca* samples, while LifeGuard mostly impacted *P. tremuloides* samples. Several of the impacted taxa at week three were also impacted in previous weeks.

**Table 2:** Summary of the effect of different preservation methods after two weeks (selected to represent an achievable shipping timeline) of incubation compared to immediate extraction, in epiphytic phyllosphere samples. Significant (*p* > 0.05) differences compared to immediate extraction are indicated as estimate and standard error in brackets while non-signficant differences are left blank. Affected parameters and corresponding preservation methods are highlighted in bold.

Species	target	Parameter	Freezing	Desiccant	Ethanol	LifeGuard	RNAlater
Picea glauca		DNA Concentration (ng/uL)	NS	NS	NS	NS	NS
Picea glauca	Bac	**Richness (# ASV)**	NS	NS	NS	**− 83.67 (27.18)**	NS
Picea glauca	Bac	Inverse Simpsons Index	NS	NS	NS	NS	NS
Picea glauca	Bac	**Shannon Index**	NS	NS	NS	**− 1.46 (0.53)**	NS
Picea glauca	Bac	PERMANOVA (p-value)	NS	NS	NS	NS	NS
Picea glauca	Bac	Betadispersion (p-value)	NS	NS	NS	NS	NS
Picea glauca	Fun	**Richness (# ASV)**	NS	NS	NS	**− 197.33 (59.88)**	**− 146.67 (59.88)**
Picea glauca	Fun	PERMANOVA (p-value)	NS	NS	NS	NS	NS
Picea glauca	Fun	Betadispersion (p-value)	NS	NS	NS	NS	NS
Populus tremuloides		**DNA Concentration (ng/uL)**	NS	**0.46 (0.09)**	NS	NS	NS
Populus tremuloides	Bac	Richness (# ASV)	NS	NS	NS	NS	NS
Populus tremuloides	Bac	Inverse Simpsons Index	NS	NS	NS	NS	NS
Populus tremuloides	Bac	**Shannon Index**	NS	NS	NS	**− 1.16 (0.51)**	NS
Populus tremuloides	Bac	PERMANOVA (p-value)	NS	NS	NS	NS	NS
Populus tremuloides	Bac	**Betadispersion (p-value)**	NS	NS	NS	**0.04**	NS
Populus tremuloides	Fun	**Richness (# ASV)**	NS	**− 109 (37.26)**	NS	**− 249 (37.26)**	**− 225.67 (37.26)**
Populus tremuloides	Fun	PERMANOVA (p-value)	NS	NS	NS	NS	NS
Populus tremuloides	Fun	**Betadispersion (p-value)**	**0.02**	NS	NS	**0**	NS

### Detection of site-level variation

**Fig. 6:**
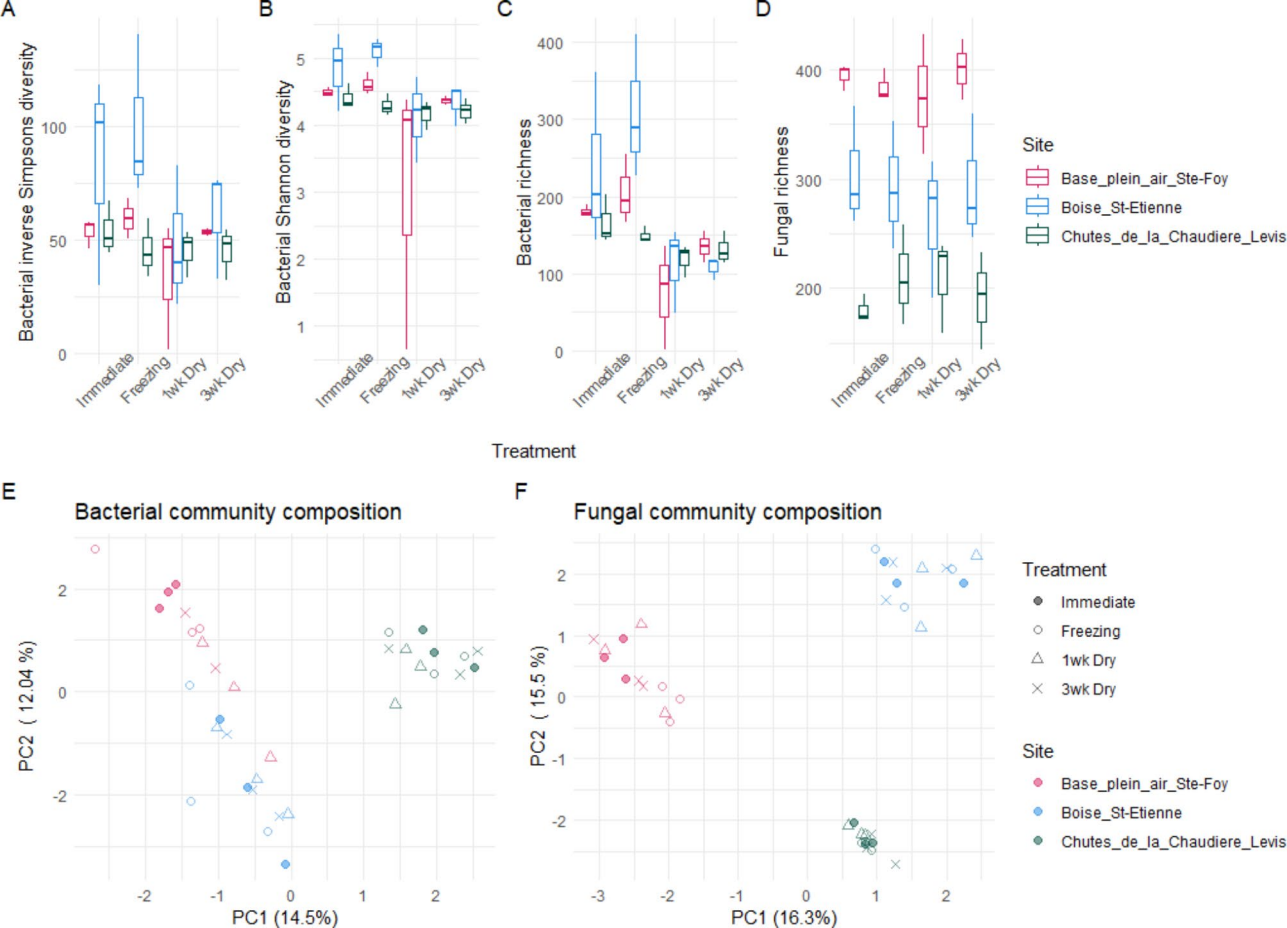
Bacterial and fungal diversity metrics and community structural PCAs for epiphytic phyllosphere samples. Panels show the following: (**A**) Bacterial inverse Simpson’s diversity; (**B**) Bacterial Shannon diversity; (**C**) Bacterial ASV richness; (**D**) Fungal ASV richness; (**E**) PCA of bacterial Aitchison distances; (**F**) PCA of fungal Aitchison distances.

Desiccation did not prevent detection of site-level effects in *P. grandidentata* phyllosphere communities. While there were significant effects of the preservation treatments on bacterial diversity metrics, detection of significant (*p* < 0.05) site-level effects for fungal and bacterial diversity, were consistent with those obtained with the immediate extraction treatment (Fig. 6A-D). Tests of community response differences to the desiccation treatment across the three sample sites showed that the site differences were a stronger influence on community structure, being the strongest drivers of the primary axes of variance for both bacterial (Fig. 6E) and fungal (Fig. 6F) communities. The preservation method did not have significant (*p* > 0.05) influences on bacterial or fungal community structure based on PERMANOVA as direct effects, or as interaction with site differences.

## Discussion

Results from this study identified dessication, as well as storage in ethanol and, RNAlater solution as relatively effective preservation treatments for short term storage of phyllosphere samples for DNA-based microbial community analyses at ambient temperature (21 °C). Generally, the *P. glauca* needles were less impacted by preservation treatments than *P. tremuloides* leaf samples, likely due to the more stable structure of the needles that was apparent with the more limited plant DNA amplified from this sample type.

Despite some impacts on DNA concentration and on the proportion of plant DNA in the *P. tremuloides* epiphytic DNA extracts, the desiccation treatment has a limited effect on microbial alpha diversity, no effect on community structure, and led to similar or lower levels of differentially abundant ASVs when compared to other preservation treatments, including freezing. Changes in the relative abundance of individual ASVs detected in the desiccation treatment samples when compared with immediate extraction mostly consisted of increases in the relative abundances of plant-associated organisms. These changes did not affect the ability to detect community compositional changes between sample types (*P. glauca* needles vs. leaves of *P. tremuloides*) or among sampling sites, as shown in a complimentary study conducted on *P. grandidentata* leaf samples from three locations.

There are several probable reasons why desiccation was a suitable preservation treatment for phyllosphere samples. Firstly, the physical structure of leaves including their surface hydrophobicity, the large surface area of leaves and needles, and presence of stomata make it easier to perform a complete desiccation of these samples^33^. Secondly, microbial communities on leaf surfaces are adapted to experience osmotic stresses^34^, making this preservation approach less likely to induce premature cell lysis in the extraction process than has been observed in other sample types^35,36^.

RNAlater and ethanol were effective at preserving DNA concentration, diversity and community structure in *P. glauca*, and ethanol was also effective at preserving these characteristics in *P. tremuloides*. For both solutions, some changes in the relative abundance of individual ASVs for both fungi and bacteria were detected, including a decrease in the relative abundance of some potential plant pathogens that occurred in the RNAlater treatment, but these changes were overall less important than those observed with the LifeGuard treatment, which was found to be an ineffective preservation solution for environmental samples in previous studies^13,37,38^. Like desiccation however, ethanol led to higher proportions of plant DNA in *P. tremuloides* DNA extracts. This was most likely because of the effect of ethanol on cell membranes which was easily observable by the green colour of the *P. tremuloides* ethanol preservation solution which was presumably from a release of chlorophyll into solution after disruption of cell and chloroplast membranes. Host DNA can interfere with the characterization of microbial communities of the phyllosphere, this was an issue with *P. tremuloides* and may be a concern with deciduous species. It may be prudent to use peptide nucleic acids (PNAs) PCR blockers^39^, targeting plant derived mitochondrial and chloroplast DNA, in future studies of phyllosphere communities to reduce the proportion of contaminant plant DNA when performing metabarcoding on desiccation and ethanol preserved broad leaf samples.

Along with the comparison of various preservation treatments, this study also provided some insights on the stability of phyllosphere samples for up to three weeks of storage. Changes in the community structure and DNA concentration of the desiccation treated samples were mostly stable over the course of three weeks. A slight delay in the water absorption was expected, but did not lead to the detection of a stronger shift in bacterial and fungal communities in week one compared to the following weeks in any of the species tested (i.e., *P. glauca*, *P. tremuloides*, *P. grandidentata*). This may indicate that microbial community changes at ambient indoor temperature are negligible in the short term in phyllosphere samples, even if the phyllosphere samples are not immediately fully dried. Similarly, dry sample storage times of three weeks or longer have also been observed to be suitable for soils^16,38,40–42^ and fecal samples^41^. The ethanol and RNAlater liquid preservation methods were also relatively stable for up to three weeks once applied. The changes to diversity and specific ASV abundances that were observed in these samples over time could be due to a delay in the activation of the preservation mechanism or to an increase of the effectiveness of this mechanism over time. Preservation solutions are immediately in contact with the exposed surfaces of samples, but may require time to access all of the sample (e.g., surfaces on the ‘inside’ of folded leaves). LifeGuard was the only liquid preservative leading to clear shifts in the microbial communities over time in phyllosphere samples. This result could have been due to inadequate inhibition of cell proliferation by this preservation solution, which was the suspected reason that community change was observed in soils preserved with this solution in Smenderovac et al.^13^.

We found that desiccation using silica gel was the most promising alternative approach to cold storage preservation methods. There were several reasons that this is the preferred method: it replicates a natural process, which places any effects into an interpretable ecological context; it had limited effects on microbial communities; it allowed for detection of site and sample effects, on community structure; and, the approach was simple and inexpensive compared to other treatments. The alternative treatments tested in this study had similar limitations to desiccation, with added logistical, cost and sampling difficulty considerations. Drying has been previously found to be an effective preservation method in soils^12,43–45^, and for plant DNA^4^. While desiccation is not the most common approach for epiphytic phyllosphere communities, some studies have used desiccation, freeze-drying and air drying as sample preservation techniques^5,46,47^ and endophytic communities are often preserved this way^4,48,49^. As drying is often employed in botanical collections^4^, this could expand the information collected from these samples. The ability to expand the reach of microbial studies to remote areas, or to recently collected herbarium samples is a worthwhile trade-off when considering the sparse number of taxa that were impacted by the desiccation treatment. Further studies will be needed however, to assess if microbial communities can be preserved for longer times and under different temperature and humidity conditions using this approach, as the current study was conducted under very specific and stable environmental conditions. It is unlikely, however, that any temperature increases will have additional effects. A study by Castano et al.^45^ supports this assumption, as they found that drying at different temperatures does not have adverse effects on DNA preservation. DNA degradation is primarily caused by enzymatic reactions, which cannot occur in the absence of water, while thermal degradation of DNA occurs at temperatures above 190 °C^50^, which is far above the temperatures expected for any field campaign. Improvements on the drying technique would be to ensure that samples are dried as quickly as possible, which may require use of additional drying agent as required.

## Conclusion

Desiccation via silica packs was found to be effective for preserving bacterial and fungal DNA in phyllosphere samples, and this method was logistically simple and cost effective. This method performed better on *P. tremuloides*, suggesting that it may be more effective approach for broadleaved species. desiccation performed comparably to the other ambient-temperature preservation methods tested on *P. glauca* samples. While some differences were detected in specific ASVs, overall community structure differences were detectable between samples from different sites and tree species. These results show that these preservation-specific changes are small and unlikely to impact detection of site, or treatment level differences. Desiccation shows promise for preserving plant-epiphytic microbial communities in situations where cold-storage is not possible. The addition of this ambient temperature alternative to cold-storage may help expand the range and number of samples we can collect from plants in logistically challenging locations such as the arctic, boreal and other remote locations.

## Electronic supplementary material

Below is the link to the electronic supplementary material.

Supplementary Material 1

## Data Availability

All sequences used in this analysis are publicly available in the NCBI Sequence Read Archive and are available under the project number PRJNA982550[https://www.ncbi.nlm.nih.gov/bioproject/PRJNA982550] with the Sample Record numbers SRR29206223 through SRR29206306 for 16 S sequences, and SRR29206861 through SRR29206944, for the ITS sequences. The RMarkdown and tabular data inputs used for creation of this manuscript are accessible on the https://github.com/Smendero/PRST repository.
