# Supplementary material for: Desiccation as a suitable alternative to cold-storage of phyllosphere samples for DNA-based microbial community analyses
Source: Sci Rep. 2025 Feb 4;15:4243. doi: 10.1038/s41598-024-82367-x (PMC11794883; doi:10.1038/s41598-024-82367-x)
Supplement: Supplementary file 1 — Supplementary Material 1 [file 41598_2024_82367_MOESM1_ESM.docx]

Supplemental Tables

Table S1: Overview of fungal and bacterial genera with at least one preservation treatment that resulted in a large (>10% relative abundance) change in a phyllosphere sample. Direction and magnitude of change that occurred for each treatment are indicated with L (Large change, >10% relative abundance change), s (small change, <10% relative abundance change), - (decrease), + (increase). Treatments without any significant change were left blank.

| Tree Species | Target | Week | Taxonomy | Freeze | Dry | EtOH | LG | RL |
| --- | --- | --- | --- | --- | --- | --- | --- | --- |
| *Picea glauca* | Bacteria | 1 | 1174 |  |  | L+, s+, L-, s- |  |  |
|  |  |  | Bradyrhizobium |  | L+ |  |  |  |
|  |  |  | Hymenobacter |  | L+ | s+ |  |  |
|  |  |  | Massilia |  | L+ | L+ |  |  |
|  |  |  | Sphingomonas |  |  | s+, L+ |  |  |
|  |  |  | Terriglobus |  |  | L+ |  |  |
|  |  | 2 | Chthonomonas |  |  | L+, s- | s- | s- |
|  |  |  | Spirosoma |  | L+ |  |  |  |
|  |  | 3 | 1174 | L-, s-, s+ | L- | s+ | L-, s- | L-, s+, s- |
|  |  |  | Deinococcus |  | L+ | s+ |  |  |
|  |  |  | Endobacter | s+ | s+ | L+ | L+ |  |
|  |  |  | Hymenobacter |  | s+, L+ | s+ |  |  |
|  |  |  | Methylobacterium |  | L+, s+ |  |  |  |
|  |  |  | Spirosoma |  | L+ |  | L- |  |
|  |  |  | WD2101 |  | L+ |  |  |  |
| *Populus tremuloides* | Bacteria | 1 | Acidiphilium |  |  | L+ |  |  |
|  |  |  | Amnibacterium |  |  | L- |  | L- |
|  |  |  | Pantoea |  |  |  |  | L+ |
|  |  |  | Sphingomonas |  |  | L+ |  |  |
|  |  | 2 | 1174 |  |  |  |  | L- |
|  |  |  | Amnibacterium |  | L- | L- |  |  |
|  |  |  | Sphingomonas |  | L- |  | L+ |  |
|  |  | 3 | Amnibacterium |  | L- |  |  | L- |
|  |  |  | Sphingomonas | L- | L- |  |  |  |
| *Picea glauca* | Fungi | 1 | Ramularia |  | L+ |  |  |  |
|  |  | 2 | Camptophora |  | L+ |  |  |  |
|  |  |  | Gibberella |  |  |  | L+ |  |
|  |  | 3 | Fusarium |  |  |  | L+ |  |
| *Populus tremuloides* | Fungi | 1 | Coniothyrium |  |  |  | L- | L- |
|  |  |  | Taphrina |  |  |  | L-, s- | s- |
|  |  | 2 | Coniothyrium |  |  |  | L- | L- |
|  |  |  | Curvibasidium |  |  | L- |  |  |
|  |  |  | Endoconidioma |  |  |  | L- |  |
|  |  |  | Endosporium |  |  | L+ |  |  |
|  |  |  | Microcyclospora |  |  |  | L- |  |
|  |  |  | Setomelanomma |  |  |  | L- |  |
|  |  |  | Sphaerulina |  |  |  | L- |  |
|  |  |  | Taphrina |  |  |  | L-, s- | L-, s- |
|  |  |  | Trichoderma |  |  |  |  | L+ |
|  |  | 3 | Coniothyrium |  |  |  | L- |  |
|  |  |  | Curvibasidium |  |  | L- |  |  |
|  |  |  | Microcyclospora |  |  |  | L- |  |
|  |  |  | Setomelanomma |  |  |  | L- | L- |
|  |  |  | Sphaerulina |  |  |  | L- |  |
|  |  |  | Taphrina |  | s- |  | L-, s- | s- |

Table S2: DNA concentration (ng/μL) ANOVA results comparing each preservation treatment to immediate extraction for each week of the study.

| Tree Species | week | parameter | Estimate | Std. Error | t value | Pr(>\|t\|) |
| --- | --- | --- | --- | --- | --- | --- |
| *Picea glauca* | 1 | Dry | -2.1741333 | 0.7574968 | -2.8701552 | 0.0166643 |
|  |  | EtOH | -1.3443333 | 0.7574968 | -1.7747050 | 0.1063374 |
|  |  | LG | -1.0433333 | 0.7574968 | -1.3773436 | 0.1984503 |
|  |  | RL | -0.6066667 | 0.7574968 | -0.8008835 | 0.4418118 |
|  | 2 | Dry | -2.1530667 | 1.7114881 | -1.2580086 | 0.2369693 |
|  |  | EtOH | -1.6200000 | 1.7114881 | -0.9465447 | 0.3661813 |
|  |  | LG | 0.9266667 | 1.7114881 | 0.5414392 | 0.6000590 |
|  |  | RL | 0.2066667 | 1.7114881 | 0.1207526 | 0.9062788 |
|  | 3 | Freeze | -1.1746667 | 0.9975796 | -1.1775167 | 0.2618158 |
|  |  | Dry | -2.1219333 | 0.9975796 | -2.1270817 | 0.0548392 |
|  |  | EtOH | -1.7597333 | 0.9975796 | -1.7640029 | 0.1031485 |
|  |  | LG | -0.1206667 | 0.9975796 | -0.1209594 | 0.9057250 |
|  |  | RL | -0.5720000 | 0.9975796 | -0.5733878 | 0.5769700 |
| *Populus tremuloides* | 1 | Dry | 0.0416000 | 0.0234037 | 1.7774965 | 0.1058566 |
|  |  | EtOH | -0.0060000 | 0.0234037 | -0.2563697 | 0.8028624 |
|  |  | LG | -0.0258667 | 0.0234037 | -1.1052382 | 0.2949380 |
|  |  | RL | -0.0258667 | 0.0234037 | -1.1052382 | 0.2949380 |
|  | 2 | Dry | 0.4598000 | 0.0923656 | 4.9780447 | 0.0005550 |
|  |  | EtOH | -0.0258667 | 0.0923656 | -0.2800466 | 0.7851493 |
|  |  | LG | -0.0258667 | 0.0923656 | -0.2800466 | 0.7851493 |
|  |  | RL | -0.0258667 | 0.0923656 | -0.2800466 | 0.7851493 |
|  | 3 | Freeze | -0.0258667 | 0.3616829 | -0.0715175 | 0.9441640 |
|  |  | Dry | 1.1308000 | 0.3616829 | 3.1264954 | 0.0087486 |
|  |  | EtOH | -0.0258667 | 0.3616829 | -0.0715175 | 0.9441640 |
|  |  | LG | 0.2838000 | 0.3616829 | 0.7846652 | 0.4478638 |
|  |  | RL | -0.0258667 | 0.3616829 | -0.0715175 | 0.9441640 |

Table S3: Diversity ANOVA results comparing Bacterial and Fungal communities from each preservation treatment to immediate extraction for each week of the study.

| Tree Species | Week | test | parameter | Estimate | Std. Error | t value | Pr(>\|t\|) |
| --- | --- | --- | --- | --- | --- | --- | --- |
| *Picea glauca* | 1 | Bacterial inverse Simpson’s diversity | Dry | -10.2791229 | 41.5161412 | -0.2475934 | 0.8094586 |
|  |  |  | EtOH | 18.9621612 | 41.5161412 | 0.4567419 | 0.6576169 |
|  |  |  | LG | -2.5819025 | 41.5161412 | -0.0621903 | 0.9516367 |
|  |  |  | RL | 57.3478992 | 41.5161412 | 1.3813398 | 0.1972571 |
|  |  | Bacterial Shannon diversity | Dry | -0.4439905 | 1.0410117 | -0.4264991 | 0.6787797 |
|  |  |  | EtOH | 0.3897192 | 1.0410117 | 0.3743658 | 0.7159483 |
|  |  |  | LG | -2.1780358 | 1.0410117 | -2.0922299 | 0.0628930 |
|  |  |  | RL | 0.5814876 | 1.0410117 | 0.5585793 | 0.5887369 |
|  |  | Bacterial ASV richness | Dry | -71.0000000 | 76.4751230 | -0.9284065 | 0.3750629 |
|  |  |  | EtOH | 32.0000000 | 76.4751230 | 0.4184367 | 0.6844723 |
|  |  |  | LG | -55.6666667 | 76.4751230 | -0.7279056 | 0.4833667 |
|  |  |  | RL | 75.6666667 | 76.4751230 | 0.9894285 | 0.3457894 |
|  |  | Fungal ASV richness | Dry | -106.6666667 | 65.6373877 | -1.6250901 | 0.1352050 |
|  |  |  | EtOH | -90.3333333 | 65.6373877 | -1.3762481 | 0.1987784 |
|  |  |  | LG | -82.0000000 | 65.6373877 | -1.2492880 | 0.2400101 |
|  |  |  | RL | -28.6666667 | 65.6373877 | -0.4367430 | 0.6715774 |
|  | 2 | Bacterial inverse Simpson’s diversity | Dry | 17.4892891 | 25.0340012 | 0.6986214 | 0.5024402 |
|  |  |  | EtOH | 22.3788193 | 25.0340012 | 0.8939370 | 0.3946320 |
|  |  |  | LG | -36.2229778 | 25.0340012 | -1.4469512 | 0.1818261 |
|  |  |  | RL | 0.4503485 | 27.9888642 | 0.0160903 | 0.9875134 |
|  |  | Bacterial Shannon diversity | Dry | 0.3386051 | 0.5255777 | 0.6442531 | 0.5354837 |
|  |  |  | EtOH | 0.3534823 | 0.5255777 | 0.6725595 | 0.5181206 |
|  |  |  | LG | -1.4644728 | 0.5255777 | -2.7864058 | 0.0211779 |
|  |  |  | RL | 0.0986719 | 0.5876138 | 0.1679197 | 0.8703592 |
|  |  | Bacterial ASV richness | Dry | 33.0000000 | 27.1820565 | 1.2140362 | 0.2556286 |
|  |  |  | EtOH | 35.0000000 | 27.1820565 | 1.2876141 | 0.2300029 |
|  |  |  | LG | -83.6666667 | 27.1820565 | -3.0780109 | 0.0131823 |
|  |  |  | RL | 29.6666667 | 30.3904631 | 0.9761834 | 0.3544808 |
|  |  | Fungal ASV richness | Dry | -37.3333333 | 59.8780242 | -0.6234897 | 0.5469198 |
|  |  |  | EtOH | -91.3333333 | 59.8780242 | -1.5253231 | 0.1581620 |
|  |  |  | LG | -197.3333333 | 59.8780242 | -3.2955886 | 0.0080734 |
|  |  |  | RL | -146.6666667 | 59.8780242 | -2.4494240 | 0.0342916 |
|  | 3 | Bacterial inverse Simpson’s diversity | Freeze | 18.8865755 | 25.8605115 | 0.7303249 | 0.4792077 |
|  |  |  | Dry | 3.8619540 | 25.8605115 | 0.1493379 | 0.8837675 |
|  |  |  | EtOH | 28.1961811 | 25.8605115 | 1.0903180 | 0.2969831 |
|  |  |  | LG | -33.7250303 | 25.8605115 | -1.3041130 | 0.2166557 |
|  |  |  | RL | 54.6599131 | 25.8605115 | 2.1136439 | 0.0561665 |
|  |  | Bacterial Shannon diversity | Freeze | 0.2928888 | 0.7555786 | 0.3876351 | 0.7050740 |
|  |  |  | Dry | 0.1435510 | 0.7555786 | 0.1899882 | 0.8524943 |
|  |  |  | EtOH | 0.4588966 | 0.7555786 | 0.6073445 | 0.5549411 |
|  |  |  | LG | -2.2400019 | 0.7555786 | -2.9646179 | 0.0118188 |
|  |  |  | RL | 0.5159344 | 0.7555786 | 0.6828335 | 0.5076806 |
|  |  | Bacterial ASV richness | Freeze | 18.3333333 | 47.4283085 | 0.3865483 | 0.7058575 |
|  |  |  | Dry | 4.3333333 | 47.4283085 | 0.0913660 | 0.9287093 |
|  |  |  | EtOH | 72.0000000 | 47.4283085 | 1.5180807 | 0.1548906 |
|  |  |  | LG | -155.6666667 | 47.4283085 | -3.2821467 | 0.0065540 |
|  |  |  | RL | 38.3333333 | 47.4283085 | 0.8082374 | 0.4346847 |
|  |  | Fungal ASV richness | Freeze | -21.6666667 | 52.4047637 | -0.4134484 | 0.6865709 |
|  |  |  | Dry | -17.3333333 | 52.4047637 | -0.3307587 | 0.7465305 |
|  |  |  | EtOH | -67.0000000 | 52.4047637 | -1.2785097 | 0.2252440 |
|  |  |  | LG | -217.6666667 | 52.4047637 | -4.1535664 | 0.0013381 |
|  |  |  | RL | -68.0000000 | 52.4047637 | -1.2975920 | 0.2188175 |
| *Populus tremuloides* | 1 | Bacterial inverse Simpson’s diversity | Dry | -10.2903751 | 5.6793161 | -1.8119039 | 0.1000910 |
|  |  |  | EtOH | 1.3645497 | 5.6793161 | 0.2402666 | 0.8149774 |
|  |  |  | LG | -11.5691408 | 5.6793161 | -2.0370658 | 0.0689850 |
|  |  |  | RL | -10.5036679 | 5.6793161 | -1.8494600 | 0.0941284 |
|  |  | Bacterial Shannon diversity | Dry | -0.5672831 | 0.4113638 | -1.3790300 | 0.1979460 |
|  |  |  | EtOH | 0.1889620 | 0.4113638 | 0.4593550 | 0.6558028 |
|  |  |  | LG | -0.8384034 | 0.4113638 | -2.0381067 | 0.0688651 |
|  |  |  | RL | -0.7954390 | 0.4113638 | -1.9336630 | 0.0819362 |
|  |  | Bacterial ASV richness | Dry | -16.3333333 | 11.0815161 | -1.4739259 | 0.1712710 |
|  |  |  | EtOH | 11.3333333 | 11.0815161 | 1.0227241 | 0.3305424 |
|  |  |  | LG | -16.6666667 | 11.0815161 | -1.5040060 | 0.1634890 |
|  |  |  | RL | -4.3333333 | 11.0815161 | -0.3910416 | 0.7039685 |
|  |  | Fungal ASV richness | Dry | -124.6666667 | 31.4451904 | -3.9645703 | 0.0026667 |
|  |  |  | EtOH | -7.3333333 | 31.4451904 | -0.2332100 | 0.8203027 |
|  |  |  | LG | -238.0000000 | 31.4451904 | -7.5687250 | 0.0000191 |
|  |  |  | RL | -174.6666667 | 31.4451904 | -5.5546385 | 0.0002425 |
|  | 2 | Bacterial inverse Simpson’s diversity | Dry | -3.3973258 | 5.2868707 | -0.6425967 | 0.5349404 |
|  |  |  | EtOH | -7.4836879 | 5.2868707 | -1.4155231 | 0.1872963 |
|  |  |  | LG | -11.5435911 | 5.2868707 | -2.1834449 | 0.0539305 |
|  |  |  | RL | -10.6941382 | 5.2868707 | -2.0227728 | 0.0706524 |
|  |  | Bacterial Shannon diversity | Dry | -0.3122541 | 0.5060725 | -0.6170145 | 0.5510142 |
|  |  |  | EtOH | -0.1930018 | 0.5060725 | -0.3813718 | 0.7109051 |
|  |  |  | LG | -1.1568189 | 0.5060725 | -2.2858760 | 0.0453305 |
|  |  |  | RL | -0.6991368 | 0.5060725 | -1.3814954 | 0.1972107 |
|  |  | Bacterial ASV richness | Dry | -8.6666667 | 13.9713994 | -0.6203149 | 0.5489252 |
|  |  |  | EtOH | 0.6666667 | 13.9713994 | 0.0477165 | 0.9628817 |
|  |  |  | LG | -23.6666667 | 13.9713994 | -1.6939367 | 0.1211424 |
|  |  |  | RL | -21.0000000 | 13.9713994 | -1.5030706 | 0.1637263 |
|  |  | Fungal ASV richness | Dry | -109.0000000 | 37.2576614 | -2.9255728 | 0.0151552 |
|  |  |  | EtOH | -75.6666667 | 37.2576614 | -2.0309022 | 0.0696994 |
|  |  |  | LG | -249.0000000 | 37.2576614 | -6.6831892 | 0.0000548 |
|  |  |  | RL | -225.6666667 | 37.2576614 | -6.0569198 | 0.0001225 |
|  | 3 | Bacterial inverse Simpson’s diversity | Freeze | -4.4470382 | 5.9118985 | -0.7522183 | 0.4664191 |
|  |  |  | Dry | -10.4927612 | 5.9118985 | -1.7748548 | 0.1012693 |
|  |  |  | EtOH | -4.8844667 | 5.9118985 | -0.8262095 | 0.4248080 |
|  |  |  | LG | -15.0588807 | 5.9118985 | -2.5472157 | 0.0255964 |
|  |  |  | RL | -11.9652924 | 5.9118985 | -2.0239340 | 0.0658276 |
|  |  | Bacterial Shannon diversity | Freeze | -0.1013041 | 0.5797524 | -0.1747368 | 0.8641999 |
|  |  |  | Dry | -0.7625617 | 0.5797524 | -1.3153230 | 0.2129800 |
|  |  |  | EtOH | -0.3697107 | 0.5797524 | -0.6377045 | 0.5356450 |
|  |  |  | LG | -1.8276745 | 0.5797524 | -3.1525087 | 0.0083360 |
|  |  |  | RL | -0.8733860 | 0.5797524 | -1.5064811 | 0.1578074 |
|  |  | Bacterial ASV richness | Freeze | 17.3333333 | 16.4643273 | 1.0527811 | 0.3131792 |
|  |  |  | Dry | -25.0000000 | 16.4643273 | -1.5184343 | 0.1548024 |
|  |  |  | EtOH | -2.0000000 | 16.4643273 | -0.1214747 | 0.9053255 |
|  |  |  | LG | -32.0000000 | 16.4643273 | -1.9435960 | 0.0757683 |
|  |  |  | RL | -15.3333333 | 16.4643273 | -0.9313064 | 0.3700534 |
|  |  | Fungal ASV richness | Freeze | -55.3333333 | 49.7984828 | -1.1111450 | 0.2882735 |
|  |  |  | Dry | -87.3333333 | 49.7984828 | -1.7537348 | 0.1049553 |
|  |  |  | EtOH | -119.3333333 | 49.7984828 | -2.3963247 | 0.0337440 |
|  |  |  | LG | -252.3333333 | 49.7984828 | -5.0670888 | 0.0002765 |
|  |  |  | RL | -205.3333333 | 49.7984828 | -4.1232849 | 0.0014123 |

Table S4: PERMANOVA results comparing Bacterial and Fungal communities from each preservation treatment to immediate extraction for each week of the study.

| Tree Species | target | week | parameter | R2 | F.value | Pr(>F) |
| --- | --- | --- | --- | --- | --- | --- |
| *Picea glauca* | Bacterial | 1 | Dry | 0.2747215 | 1.1363420 | 0.3 |
|  |  |  | EtOH | 0.2777790 | 1.5384709 | 0.1 |
|  |  |  | LG | 0.2218451 | 1.1403645 | 0.2 |
|  |  |  | RL | 0.2072282 | 1.0455879 | 0.5 |
|  |  | 2 | Dry | 0.2514539 | 1.3436924 | 0.1 |
|  |  |  | EtOH | 0.2363610 | 1.2380772 | 0.1 |
|  |  |  | LG | 0.2529906 | 1.3546850 | 0.1 |
|  |  |  | RL | 0.2952069 | 1.2565682 | 0.1 |
|  |  | 3 | Dry | 0.2963468 | 1.6846183 | 0.1 |
|  |  |  | EtOH | 0.2549391 | 1.3686890 | 0.1 |
|  |  |  | Freeze | 0.2589460 | 1.3977176 | 0.1 |
|  |  |  | LG | 0.3024796 | 1.7345996 | 0.1 |
|  |  |  | RL | 0.2400396 | 1.2634319 | 0.1 |
|  | Fungal | 1 | Dry | 0.3076033 | 1.3327765 | 0.1 |
|  |  |  | EtOH | 0.2620461 | 1.4203930 | 0.1 |
|  |  |  | LG | 0.2281670 | 1.1824684 | 0.2 |
|  |  |  | RL | 0.2017895 | 1.0112097 | 0.5 |
|  |  | 2 | Dry | 0.2409112 | 1.2694758 | 0.1 |
|  |  |  | EtOH | 0.2534922 | 1.3582832 | 0.1 |
|  |  |  | LG | 0.3009594 | 1.7221284 | 0.1 |
|  |  |  | RL | 0.2356436 | 1.2331610 | 0.1 |
|  |  | 3 | Dry | 0.2862842 | 1.6044718 | 0.1 |
|  |  |  | EtOH | 0.2595457 | 1.4020893 | 0.1 |
|  |  |  | Freeze | 0.2465000 | 1.3085598 | 0.1 |
|  |  |  | LG | 0.3486497 | 2.1410886 | 0.1 |
|  |  |  | RL | 0.2270987 | 1.1753054 | 0.1 |
| *Populus tremuloides* | Bacterial | 1 | Dry | 0.2195798 | 1.1254437 | 0.1 |
|  |  |  | EtOH | 0.2240318 | 1.1548505 | 0.2 |
|  |  |  | LG | 0.2510898 | 1.3410945 | 0.1 |
|  |  |  | RL | 0.2493022 | 1.3283758 | 0.2 |
|  |  | 2 | Dry | 0.2025220 | 1.0158125 | 0.6 |
|  |  |  | EtOH | 0.1930165 | 0.9567310 | 0.7 |
|  |  |  | LG | 0.2225153 | 1.1447955 | 0.1 |
|  |  |  | RL | 0.2333644 | 1.2176028 | 0.1 |
|  |  | 3 | Dry | 0.2117871 | 1.0747713 | 0.3 |
|  |  |  | EtOH | 0.2114462 | 1.0725771 | 0.1 |
|  |  |  | Freeze | 0.1975562 | 0.9847726 | 0.5 |
|  |  |  | LG | 0.2608842 | 1.4118715 | 0.1 |
|  |  |  | RL | 0.2303071 | 1.1968779 | 0.1 |
|  | Fungal | 1 | Dry | 0.2540397 | 1.3622158 | 0.1 |
|  |  |  | EtOH | 0.2510337 | 1.3406941 | 0.1 |
|  |  |  | LG | 0.4075003 | 2.7510585 | 0.1 |
|  |  |  | RL | 0.2734467 | 1.5054459 | 0.1 |
|  |  | 2 | Dry | 0.2461203 | 1.3058860 | 0.1 |
|  |  |  | EtOH | 0.2672395 | 1.4588098 | 0.1 |
|  |  |  | LG | 0.4159950 | 2.8492565 | 0.1 |
|  |  |  | RL | 0.3480011 | 2.1349794 | 0.1 |
|  |  | 3 | Dry | 0.2256703 | 1.1657582 | 0.1 |
|  |  |  | EtOH | 0.2480641 | 1.3196022 | 0.1 |
|  |  |  | Freeze | 0.2295434 | 1.1917264 | 0.1 |
|  |  |  | LG | 0.4262012 | 2.9710852 | 0.1 |
|  |  |  | RL | 0.3313676 | 1.9823607 | 0.1 |

Table S5: Betadispersal results comparing each treatment level to immediate extraction for each week of the study.

| Tree Species | target | week | parameter | Mean.Sq | F.value | Pr(>F) |
| --- | --- | --- | --- | --- | --- | --- |
| *Picea glauca* | Bacterial | 1 | Dry | 0.2917245 | 0.2214296 | 0.6700513 |
|  |  |  | EtOH | 0.0490353 | 0.0222829 | 0.8885608 |
|  |  |  | LG | 1.3405549 | 0.1101253 | 0.7566614 |
|  |  |  | RL | 11.6227378 | 2.4480499 | 0.1927203 |
|  |  | 2 | Dry | 0.1764456 | 0.1703760 | 0.7009437 |
|  |  |  | EtOH | 9.9822611 | 2.8837180 | 0.1647114 |
|  |  |  | LG | 1.1266612 | 0.4011407 | 0.5609056 |
|  |  |  | RL | 0.0018744 | 0.0014284 | 0.9722264 |
|  |  | 3 | Dry | 1.9370757 | 1.5740107 | 0.2779321 |
|  |  |  | EtOH | 14.2949615 | 5.8277276 | 0.0732315 |
|  |  |  | Freeze | 0.9299000 | 0.4621664 | 0.5339219 |
|  |  |  | LG | 11.6588139 | 0.8199722 | 0.4163999 |
|  |  |  | RL | 0.0121801 | 0.0102825 | 0.9241104 |
|  | Fungal | 1 | Dry | 8.2135017 | 4.5099858 | 0.1237508 |
|  |  |  | EtOH | 6.8680380 | 4.2347471 | 0.1087173 |
|  |  |  | LG | 0.1215922 | 0.0175899 | 0.9008927 |
|  |  |  | RL | 1.0412612 | 0.1080557 | 0.7588583 |
|  |  | 2 | Dry | 8.8108346 | 6.1604352 | 0.0680634 |
|  |  |  | EtOH | 0.4395943 | 0.2013940 | 0.6768364 |
|  |  |  | LG | 14.1944876 | 1.4778713 | 0.2909472 |
|  |  |  | RL | 2.7417253 | 1.9900464 | 0.2311606 |
|  |  | 3 | Dry | 3.0330729 | 1.9578965 | 0.2343087 |
|  |  |  | EtOH | 2.0252848 | 0.6596121 | 0.4622646 |
|  |  |  | Freeze | 2.3808519 | 0.9207683 | 0.3916127 |
|  |  |  | LG | 23.0186159 | 1.0899632 | 0.3554184 |
|  |  |  | RL | 5.5538000 | 1.6571084 | 0.2674311 |
| *Populus tremuloides* | Bacterial | 1 | Dry | 0.1416005 | 0.0842676 | 0.7860229 |
|  |  |  | EtOH | 1.0075273 | 1.2156051 | 0.3320992 |
|  |  |  | LG | 9.3059867 | 8.1582541 | 0.0461030 |
|  |  |  | RL | 5.6532852 | 7.7569227 | 0.0495582 |
|  |  | 2 | Dry | 0.3136277 | 0.1905116 | 0.6850173 |
|  |  |  | EtOH | 3.0309475 | 0.6496675 | 0.4654201 |
|  |  |  | LG | 7.0352770 | 8.7781497 | 0.0414394 |
|  |  |  | RL | 4.5736757 | 2.8112698 | 0.1689108 |
|  |  | 3 | Dry | 1.9681843 | 1.6870308 | 0.2638069 |
|  |  |  | EtOH | 4.7027075 | 4.5345695 | 0.1002696 |
|  |  |  | Freeze | 17.4212616 | 6.0849941 | 0.0691857 |
|  |  |  | LG | 1.0501091 | 0.8313370 | 0.4134659 |
|  |  |  | RL | 6.5517784 | 6.3238472 | 0.0657252 |
|  | Fungal | 1 | Dry | 2.7077151 | 7.6690482 | 0.0503669 |
|  |  |  | EtOH | 0.7524797 | 1.6759040 | 0.2651452 |
|  |  |  | LG | 36.6408077 | 47.6053912 | 0.0023139 |
|  |  |  | RL | 7.8214829 | 21.4340897 | 0.0098094 |
|  |  | 2 | Dry | 0.1096900 | 0.0314540 | 0.8678499 |
|  |  |  | EtOH | 0.4357450 | 0.5159251 | 0.5123039 |
|  |  |  | LG | 72.1855398 | 46.0011072 | 0.0024669 |
|  |  |  | RL | 23.9669820 | 5.4814074 | 0.0792707 |
|  |  | 3 | Dry | 1.3157707 | 0.7990074 | 0.4219122 |
|  |  |  | EtOH | 0.7791053 | 0.1754350 | 0.6968392 |
|  |  |  | Freeze | 1.0080584 | 13.0844740 | 0.0224119 |
|  |  |  | LG | 59.4642885 | 32.6266607 | 0.0046462 |
|  |  |  | RL | 23.0721813 | 9.3377871 | 0.0378140 |
